# Assessing the utility and challenges for implementation of a risk prediction system: a usability study with hospital pharmacists

**DOI:** 10.1186/s40780-025-00499-2

**Published:** 2025-10-27

**Authors:** Keisuke Ikegami, Masami Tsuchiya, Hayato Kizaki, Shungo Imai, Osamu Yasumuro, Chiaki Sato, Yukiyoshi Fujita, Ryohkan Funakoshi, Satoko Hori

**Affiliations:** 1 Keio University Faculty of Pharmacy/Graduate School of Pharmaceutical Sciences, 1-5-30 Shibakoen, Minato-ku, Tokyo, 105-8512 Japan; 2 Department of Pharmacy, Kameda General Hospital, 929 Higashi-cho, Kamogawa, Chiba 296-8602 Japan; 3 Department of Pharmacy, Miyagi Cancer Center, 47-1 Nodayama, Medeshimashiote, Natori, Miyagi 981-1293 Japan; 4 Department of Pharmacy, Gunma Prefectural Cancer Center, 617-1 Takahayashi Nishicho, Ota, Gunma 373-8550 Japan

**Keywords:** Clinical prediction model, Scoring system, Clinical implementation, End-user evaluation, Questionnaire survey, Denosumab, Hypocalcemia

## Abstract

**Background:**

The clinical implementation of prediction models can face important barriers, particularly regarding user-friendliness and interpretability for healthcare professionals. We recently developed and externally validated a risk prediction model for denosumab-induced hypocalcemia. The present study aimed to evaluate the model’s utility and identify challenges for its clinical implementation through pilot testing conducted by hospital pharmacists.

**Methods:**

A paper-format prediction model was distributed to pharmacists at Kameda General Hospital, Miyagi Cancer Center, and Gunma Prefectural Cancer Center. Participants trialed the model outside their routine workflow by applying it to data from patients scheduled to receive their first dose of denosumab. A subsequent questionnaire survey, available in paper and electronic formats, was conducted to gather feedback on the model’s utility and limitations.

**Results:**

A total of 49 responses were obtained, predominantly from pharmacists in their 20 s and 30 s with diverse professional responsibilities. The model was positively evaluated in terms of its clear target population, predicted outcome (47/49, 95.9%), and simple, straightforward nature (47/49, 95.9%). However, some participants provided neutral feedback on its ease of use (10/49, 20.4%). While its potential as an auxiliary tool for risk prediction was acknowledged, there were also some neutral views on its practical utility. One concern was the inconvenience of implementing a paper-format tool in clinical environments that primarily operate on electronic health records.

**Conclusions:**

The paper-format prediction model was positively evaluated by frontline pharmacists, especially for its clear and straightforward nature. As anticipated, however, limitations such as manual data input and paper-based format were identified by some participants. Integration into electronic health records and broader clinical validation will be necessary to advance the clinical application of this prediction model and ensure real-world applicability.

**Supplementary Information:**

The online version contains supplementary material available at 10.1186/s40780-025-00499-2.

## Introduction

Evidence-based clinical prediction models can be valuable tools for supporting decision-making by healthcare professionals and for optimizing individualized patient care. Although numerous high-performance prediction systems have been developed, their integration into routine clinical practice remains limited [1, 2]. Key barriers include the lack of user-friendliness and concerns regarding model interpretability [1–3]. Therefore, evaluation by end-users is essential to ensure the effective implementation and practical usability of prediction models in clinical settings.

We recently developed and externally validated a scoring system to predict grade ≥ 2 denosumab-induced hypocalcemia within 28 days in patients with cancer and bone metastases [4, 5]. This system was developed as part of a series of studies focused on the importance of validating model performance on a facility-by-facility basis. The model utilizes carefully selected variables to enhance interpretability and minimize inter-facility variability in performance. To date, the model has demonstrated high values of receiver operating characteristic-area under the curve (ROC-AUC), ranging from 0.812 to 0.856 across three general and cancer hospitals in Japan.

Although we have undertaken a series of studies to obtain evidence to underpin the practical implementation of clinical prediction models—using denosumab-induced hypocalcemia as a representative case—evaluation from the perspective of end-users in actual clinical settings has not yet been conducted. Incorporating the views of frontline healthcare professionals is an important step toward practical implementation. Therefore, the present case study aimed to assess the clinical utility of our prediction model and also to identify potential challenges in applying the model from the perspective of hospital pharmacists.

## Methods

### Study design and participants

This study was conducted between September 2024 and June 2025 using a self-administered, anonymous questionnaire. The questionnaire was distributed in both paper and electronic formats, and responses were collected either via Google Forms or by scanning submitted documents. Participants were pharmacists working at Kameda General Hospital (Chiba), Miyagi Cancer Center (Miyagi), and Gunma Prefectural Cancer Center (Gunma). These institutions are estimated to have no fewer than 40 new cases of denosumab administration annually in patients with bone metastases from solid tumors. This model is specifically designed as an adjunctive tool for the pharmaceutical management of the risk of denosumab-induced hypocalcemia. Consequently, the model’s evaluation was limited to pharmacists in this study. The characteristics and denosumab usage status of Kameda General Hospital and Miyagi Cancer Center have been reported elsewhere [4], while those of Gunma Prefectural Cancer Center were reported separately [5].

### Clinical prediction model

In this study, we utilized a previously developed and validated prediction model [4, 5]. This scoring model estimates the risk of denosumab-induced hypocalcemia based on three pretreatment laboratory parameters: corrected calcium (< 8.8 mg/dL: 3 point, 8.8 to < 9.2: 2 point, ≥ 9.2: 0 point), albumin (< 3.5 g/dL: 1 point, ≥ 3.5: 0 point), and alkaline phosphatase (< 113 U/L: 0 point, 113 to < 195: 1 point, ≥ 195: 2 point). The model demonstrated its highest discriminant performance when a total score of ≥ 2 points was used to define high-risk patients.

### Model use and questionnaire survey

In this study, participants used a paper-format prediction model, which was prepared for research purposes (Supplementary Material). After reviewing an explanatory document provided by the collaborating investigator at each participating institution, participants trialed the model outside their regular clinical workflow by applying it to clinical data from patients scheduled to receive their first dose of denosumab. In accordance with the study protocol, the results generated by the model were not used to inform or alter any clinical decisions.

Subsequently, a questionnaire survey was conducted to obtain feedback regarding the model’s utility and potential limitations. Responses to the questionnaire were solicited by the same investigator at each participating institution. The survey was developed with reference to previous studies [6–8] and consisted of three components: (A) respondent demographics (sex, age group, years of professional experience as a hospital pharmacist, job responsibilities), (B) perceived usefulness of the prediction model (model’s interpretability, user-friendliness and clinical utility), and (C) identified challenges associated with the model (model’s potential risk). A total of 13 questions were included in components B and C, primarily using a 5-point Likert scale. The contents of the survey are provided in detail in Tables 1, 2 and 3.

### Descriptive analysis of survey data

The quantitative data were analyzed by calculating response frequencies. For the qualitative data, a thematic analysis was conducted. Two independent raters (a PhD candidate in pharmacy and a clinical pharmacist with 17 years of experience, both licensed pharmacists) coded the free-text responses, and inter-rater reliability was determined using Cohen’s kappa. Disagreements in coding were resolved by consensus between the raters.

### Ethical considerations

Formal written consent was not collected; participation in the study was considered to imply consent. The explanatory document clearly stated that participation was voluntary and that no disadvantage would result from choosing not to respond. The survey was anonymous, and personal identification was not possible after submission; thus, withdrawal of participation was not feasible once responses had been submitted. Since participants might have made notes of patient data when trialing the prediction model, all completed paper forms were securely discarded within the pharmacy department in a manner that ensured complete irretrievability. The study protocol was reviewed and approved by the Ethics Committee of the Keio University Faculty of Pharmacy on September 2, 2024 (Approval No. 240902-1), and the study was conducted in accordance with the Declaration of Helsinki.

## Results

### Participant demographics

In total, 49 responses were obtained: 30 from Kameda General Hospital (response rate: 100%), 11 from Miyagi Cancer Center (response rate: 40.7%), and 8 from Gunma Prefectural Cancer Center (response rate: 57.1%). There was no marked difference in gender among the respondents at different institutions, and responses were obtained from a wide range of age groups, predominantly in the 20 s and 30 s. While respondents from Kameda General Hospital were predominantly from younger age groups with less professional experience, their job responsibilities varied widely (Table 1). Although the majority of respondents were engaged in dispensing work, they were also responsible for prescription review and thus are representative of the model’s intended user group.

**Table 1:** (A) Participant demographics

		Total (*N* = 49)	Kameda General Hospital (*n* = 30)	Miyagi Cancer Center (*n* = 11)	Gunma Prefectural Cancer Center (*n* = 8)
Female, n (%)		26 (53.1)	16 (53.3)	5 (45.5)	5 (62.5)
Age group, n (%)	20–29	22 (44.9)	20 (66.7)	2 (18.2)	0
	30–39	15 (30.6)	10 (33.3)	3 (27.3)	2 (25.0)
	40–49	9 (18.4)	0	5 (45.5)	4 (50.0)
	50–59	1 (2.0)	0	0	1 (12.5)
	60 +	2 (4.1)	0	1 (9.1)	1 (12.5)
Years of professional experience as a hospital pharmacist, n (%)	< 1 year	0	0	0	0
	1—4 years	22 (44.9)	20 (66.7)	2 (18.2)	0
	5—9 years	14 (28.6)	10 (33.3)	2 (18.2)	2 (25.0)
	10—14 years	3 (6.1)	0	2 (18.2)	1 (12.5)
	15—19 years	3 (6.1)	0	2 (18.2)	1 (12.5)
	≥ 20 years	7 (14.3)	0	3 (27.3)	4 (50.0)
Job responsibilities (multiple answers allowed)	Inpatient dispensing	18 (36.7)	10 (33.3)	3 (27.3)	5 (62.5)
	Outpatient dispensing	14 (28.6)	7 (23.3)	2 (18.2)	5 (62.5)
	Sterile compounding (injections)	15 (30.6)	5 (16.7)	6 (54.5)	4 (50.0)
	Chemotherapy compounding	24 (49.0)	19 (63.3)	2 (18.2)	3 (37.5)
	Drug information services	1 (2.0)	0	0	1 (12.5)
	Medication management	3 (6.1)	0	2 (18.2)	1 (12.5)
	Narcotics management	2 (4.1)	0	0	2 (25.0)
	Ward pharmacy services	19 (38.8)	10 (33.3)	5 (45.5)	4 (50.0)
	Other	6 (12.2)	0	3 (27.3)	3 (37.5)

Values are presented as number of respondents (%)

### Perceived usefulness and challenges of the prediction model

The responses to the questionnaire items regarding the usefulness of the prediction model are summarized in Table 2. In the evaluation of the model’s interpretability, the clarity of its purpose (Q1) was viewed positively by most respondents. In contrast, as regards the ease of interpreting the figure (Q2), approximately 20% of responses were neutral. Regarding user-friendliness, while about 20% of respondents remained neutral on whether the model could be used quickly (Q3), the simplicity and straightforwardness of the calculation process itself (Q4) received largely positive feedback. In the assessment of the model’s clinical usefulness, its utility for implementation in the clinical workflow (Q5) was met with responses ranging from neutral to agreement. Among the respondents who agreed with Q5, many indicated in their open-ended answers (Q6) that the model would be useful not only for prescription audits, such as when checking laboratory values, but also for treatment monitoring, including ward duties. Inter-rater reliability was high, with Cohen’s kappa exceeding 0.80 across all categories. Furthermore, the question regarding the model’s significance as a valuable adjunctive tool in clinical management (Q7) also received generally positive responses.

**Table 2:** (B) Perceived usefulness of the prediction model

	Total (*N* = 49)	Kameda General Hospital (*n* = 30)	Miyagi Cancer Center (*n* = 11)	Gunma Prefectural Cancer Center (*n* = 8)
Regarding Interpretability				
Q1. The model’s purpose (i.e., the target patient population and the predicted outcome) is clear and easy to understand				
Strongly agree	12 (24.5)	6 (20.0)	4 (36.4)	2 (25.0)
Agree	35 (71.4)	24 (80.0)	5 (45.5)	6 (75.0)
Neither agree nor disagree	2 (4.1)	0	2 (18.2)	0
Disagree	0	0	0	0
Strongly disagree	0	0	0	0
Q2. The figure associated with the model is easy to interpret				
Strongly agree	12 (24.5)	6 (20.0)	4 (36.4)	2 (25.0)
Agree	27 (55.1)	18 (60.0)	4 (36.4)	5 (62.5)
Neither agree nor disagree	10 (20.4)	6 (20.0)	3 (27.3)	1 (12.5)
Disagree	0	0	0	0
Strongly disagree	0	0	0	0
Regarding User-friendliness				
Q3. Calculating a patient’s score and predicting their risk using the model can be performed quickly				
Strongly agree	26 (53.1)	18 (60.0)	6 (54.5)	2 (25.0)
Agree	13 (26.5)	6 (20.0)	2 (18.2)	5 (62.5)
Neither agree nor disagree	10 (20.4)	6 (20.0)	3 (27.3)	1 (12.5)
Disagree	0	0	0	0
Strongly disagree	0	0	0	0
Q4. The process for calculating scores and predicting risks with the model is simple and straightforward				
Strongly agree	26 (53.1)	18 (60.0)	6 (54.5)	2 (25.0)
Agree	21 (42.9)	12 (40.0)	3 (27.3)	6 (75.0)
Neither agree nor disagree	2 (4.1)	0	2 (18.2)	0
Disagree	0	0	0	0
Strongly disagree	0	0	0	0
Regarding Clinical Utility				
Q5. The prediction model is useful for assessing the risk of hypocalcemia in target patients as part of the clinical workflow				
Strongly agree	4 (8.2)	0	4 (36.4)	0
Agree	31 (63.3)	24 (80.0)	3 (27.3)	4 (50.0)
Neither agree nor disagree	14 (28.6)	6 (20.0)	4 (36.4)	4 (50.0)
Disagree	0	0	0	0
Strongly disagree	0	0	0	0
Q6. If you answered “Strongly Agree” or “Agree” to the question above, at which point in the clinical workflow do you believe this model could be best utilized? (Open-ended)				
Prescription Review	19	11	5	3
Laboratory Data Review	5	4	1	0
Treatment Monitoring	13	9	2	2
Q7. The prediction model serves as a valuable adjunctive tool in the clinical management of hypocalcemia associated with denosumab therapy				
Strongly agree	9 (18.4)	6 (20.0)	3 (27.3)	0
Agree	28 (57.1)	18 (60.0)	4 (36.4)	6 (75.0)
Neither agree nor disagree	12 (24.5)	6 (20.0)	4 (36.4)	2 (25.0)
Disagree	0	0	0	0
Strongly disagree	0	0	0	0
Q8. If you answered “Disagree” or “Strongly Disagree” to the question above, what specific issues or limitations with the model led to your response? (Open-ended)				
	N/A	N/A	N/A	N/A
Q9. If you have any further comments or suggestions for improvement, please describe them below. (Optional)				
		N/A	-A system integrated into the EHR that automatically assesses risk from lab values (e.g., by displaying the risk score alongside other test results) - The target patient population for the model should be more clearly defined - Adding more white space to the score calculation diagram would improve its readability	- The model should be validated through actual clinical implementation to gather user feedback - A tool that provides a risk score upon entering lab values would be useful

Values are presented as number of respondents (%). The free-text responses in Q6 were coded into only three categories

Table 3 shows the responses to the survey questions on the challenges of the prediction model. Regarding the potential risks of the model, no respondents reported encountering situations that caused confusion or uncertain clinical judgments (Q10); however, approximately 30% of the responses were neutral on this point. Concerning the increase in workload from implementing the model (Q12), the feedback indicated that it would impose a slight to moderate burden. Finally, in the optional feedback on areas for improvement (Q13), respondents pointed out a need to improve the figure’s design and noted a lack of clarity regarding calcium value correction.

**Table 3:** (C) Identified challenges associated with the model

	Total (*N* = 49)	Kameda General Hospital (*n* = 30)	Miyagi Cancer Center (*n* = 11)	Gunma Prefectural Cancer Center (*n* = 8)
Regarding Potential Risks				
Q10. While using the prediction model, I encountered situations that caused confusion or led to uncertain clinical judgments				
Strongly agree	0	0	0	0
Agree	0	0	0	0
Neither agree nor disagree	15 (30.6)	6 (20.0)	4 (36.4)	5 (62.5)
Disagree	32 (65.3)	24 (80.0)	6 (54.5)	2 (25.0)
Strongly disagree	2 (4.1)	0	1 (9.1)	1 (12.5)
Q11. If you answered “Strongly Agree” or “Agree” to the question above, what specific aspects of the model caused this confusion or uncertainty? (Open-ended)				
	N/A	N/A	N/A	N/A
Q12. To what extent would integrating this prediction model (in its current paper-based format) into your clinical workflow affect your workload? Please consider the overall burden in terms of time and effort				
A very high burden	0	0	0	0
A moderate burden	4 (8.2)	0	2 (18.2)	2 (25.0)
Neutral	9 (18.4)	6 (20.0)	2 (18.2)	1 (12.5)
A slight burden	36 (73.5)	24 (80.0)	7 (63.6)	5 (62.5)
No burden at all	0	0	0	0
Q13. Please describe any aspects of the model’s explanation, design, or layout that were unclear or difficult to understand. (Optional)				
		- The score’s visibility was poor because it was embedded directly on the horizontal axis. It was also unclear that the pre-corrected calcium value could be used when no correction is necessary	- The requirement for a “corrected calcium value” was unclear; I almost used the pre-correction value by mistake	N/A

Values are presented as number of respondents (%)

## Discussion

This study aimed to evaluate the clinical usability of a previously validated risk prediction model for denosumab-induced hypocalcemia from the perspective of hospital pharmacists. Although the model was presented in a paper format for research purposes, obtaining feedback from frontline healthcare professionals represents a critical step in implementing prediction models in routine clinical practice. We believe that this study serves as an important example highlighting this critical step.

The respondent pool consisted predominantly of individuals in their 20 s to 30 s. While there were variations in age distribution across the facilities, evaluations of the model’s clinical utility and perceived challenges were largely consistent. In terms of interpretability, the majority of respondents expressed a positive perception (Q1, Q2). However, 20.4% of respondents reported a neutral stance regarding the interpretability of the model’s figure (Q2). This finding highlights the need to improve the color scheme of the logistic regression curve and its text, as well as the use of white space.

Regarding the model’s user-friendliness, the feedback was predominantly positive (Q3, Q4), which can be attributed to the emphasis placed on this aspect during development. However, some respondents provided neutral feedback on the model’s ability to enable rapid risk prediction (Q3). This was likely due to the paper format used in this study, which required users to manually look up lab values in the electronic health records (EHRs) and transcribe them into the model. For clinical implementation, integrating such prediction tools directly into EHRs is the preferred approach [2, 9, 10]. Therefore, it is anticipated that the evaluation of this aspect—time required for assessment—would improve substantially with a digital, EHR-integrated version of the model.

In terms of clinical utility as assessed in Q5 and Q7, 71.4% to 75.5% of respondents reported a positive stance; however, a proportion of respondents remained neutral. A potential barrier to adoption is likely the challenge of implementing a paper-format tool in clinical environments that operate on EHRs. Consequently, as indicated by the feedback in Q9, integrating the model into the EHR to enable automated risk calculation would likely improve its perceived usefulness. Indeed, models integrated into the EHR have been demonstrated to guide healthcare providers toward more evidence-based clinical practices [11]. Additionally, feedback on potential use cases (Q6) included suggestions for extending the model’s use for continuous risk assessment. Since the current model is validated only for risk assessment at the initial denosumab administration, future research should examine its applicability to subsequent treatment courses.

Regarding the survey on potential risks, no respondent reported experiencing confusion or uncertainty in clinical judgment while using the model, based on responses to Q10. However, it should be acknowledged that approximately 30% of respondents selected a neutral response. Notably, in the free-text responses to Q13, some participants mentioned uncertainty about whether to input corrected or uncorrected calcium values into the model. This ambiguity was considered a potential source of confusion and represents an important area for future model refinement. Furthermore, as expected, responses indicated that integrating a paper-format model into existing workflows is perceived as a burden (Q12), reinforcing the conclusion that direct EHR integration would be the optimal solution.

For future research on the clinical application of this predictive model, a comparative trial against a non-use control group would be desirable after reflecting feedback obtained [3, 12]. However, trials that assess the actual impact on patient care are rarely conducted [13], and it is important to consider more feasible approaches. These include “before-after” impact analyses, which compare periods before and after the model’s implementation [12, 13], or “on–off” impact analyses that alternate between periods with and without the model’s use [13].

One of the limitations of this study may be lack of generalizability, as the model was focused on a single clinical event. Therefore, the findings may not necessarily be directly applicable to other prediction models. Nevertheless, presenting this case study evaluating clinical utility and implementation challenges provides insights that may inform broader efforts in prediction model research. Another limitation is the potential for positive response bias, as the participants were from institutions previously involved in the development and evaluation of this model. This contextual factor may have influenced the responses of participants.

In conclusion, this study assessed the usability and challenges of a denosumab-induced hypocalcemia risk prediction model from the perspective of hospital pharmacists. The model’s clear and straightforward design was positively evaluated; however, limitations such as a manual data input and paper-based format were identified, indicating that an electronic format would be preferable for seamless integration into the clinical workflow. Despite these limitations, this end-user evaluation should be helpful to guide future research on the clinical implementation of prediction models. Integration into electronic health records and broader clinical validation, such as increasing the number of study sites and extending the scope to other healthcare professionals, will be needed to promote real-world applicability.

## Supplementary Information

Supplementary Material 1.

## Data Availability

The datasets generated during the current study are available from the corresponding author on reasonable request.

## Abbreviations

EHRs: Electronic health records
ROC-AUC: Receiver operating characteristic-area under the curve
