# Supplementary material for: Assessing the utility and challenges for implementation of a risk prediction system: a usability study with hospital pharmacists
Source: J Pharm Health Care Sci. 2025 Oct 27;11:96. doi: 10.1186/s40780-025-00499-2 (PMC12560321; doi:10.1186/s40780-025-00499-2)

The original version of this document was written in Japanese.

Study Title:

Assessing the Utility and Challenges for Implementation of a Risk Prediction System: A Usability Study with Hospital Pharmacists

Pilot Testing Instructions for Hospital Pharmacists:

- The aim of this study is to identify the utility and challenges of the risk prediction model for denosumab-induced hypocalcemia (see next page) through pilot testing.
- Please do not incorporate the prediction results obtained from using the model into clinical decision-making.
- Regarding the "Target Patients" in the explanation of the prediction model on the next page, the model was originally designed to predict risk **prior to denosumab administration**, as indicated. However, since this study asks participants to test the model at any point outside of routine workflows, there may be cases where it is applied **after denosumab administration**. In such instances, please use the model with the assumption of a pre-administration scenario in mind.

The prediction model is shown on the next page (on the reverse side).

The original version of this document was written in Japanese.

Please review the following information before using this prediction model:

Target Patients:

Patients with bone metastases from solid tumors who are scheduled to receive their first subcutaneous dose of denosumab 120 mg (RANMARK® Subcutaneous Injection 120 mg).

What This Model Predicts:

The probability (risk) of developing grade 2 ≥ hypocalcemia* within 28 days.

Note (Supplemental Information):

This model was developed based on an analysis of a patient population receiving two tablets per day of DENOTAS® Chewable Combination Tablets. Therefore, the predicted risk applies to patients who are concomitantly receiving DENOTAS®.

*Corrected Ca value <8.0 mg/dL (CTCAE ver. 5.0)

1. Circle the score corresponding to the range of each laboratory parameter, and calculate the total score.

Corrected Ca value (mg/dL) Low — 3 — 8.7 | 8.8 — 2 — 9.1 | 9.2 — 0 → High

Alb value (g/dL) Low — 1 — 3.4 | 3.5 — 0 → High

ALP value (U/L, IFCC) Low — 0 — 112 | 113 — 1 — 194 | 195 — 2 → High

Corrected Ca value (mg/dL) =
Measured Ca value (mg/dL) + (4 - Alb value (g/dL))
(if Alb value < 4 d/dL)

Total score : _______

2. Determine the probability (risk) of hypocalcemia based on the total score.

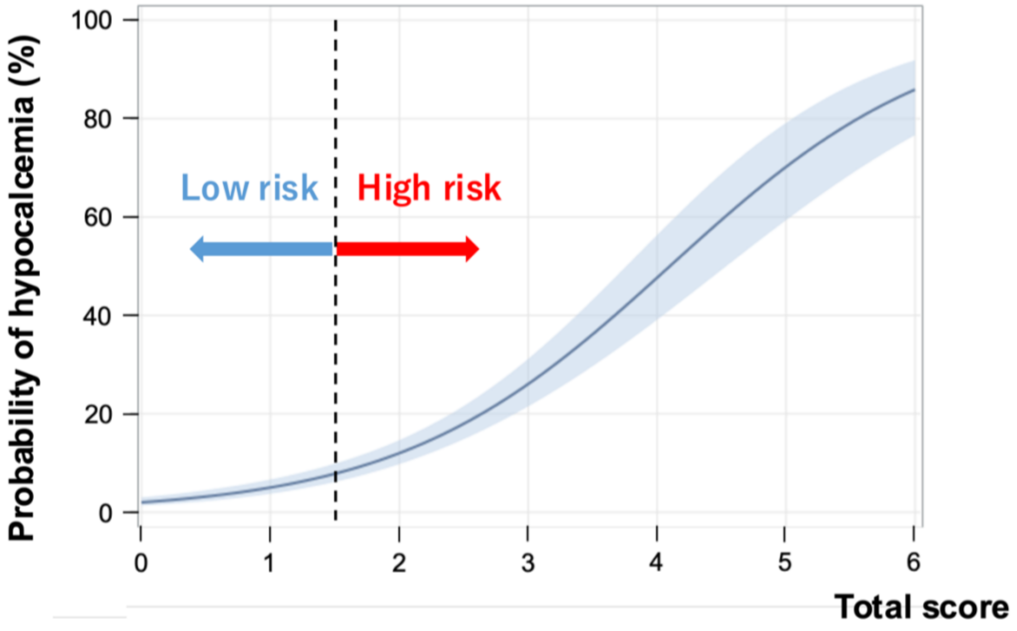

Supplement: Supplementary file 1 — Supplementary Material 1. [file 40780_2025_499_MOESM1_ESM.pdf]
